# Curcumin Protects Human Keratinocytes against Inorganic Arsenite-Induced Acute Cytotoxicity through an NRF2-Dependent Mechanism

**DOI:** 10.1155/2013/412576

**Published:** 2013-04-21

**Authors:** Rui Zhao, Bei Yang, Linlin Wang, Peng Xue, Baocheng Deng, Guohua Zhang, Shukun Jiang, Miao Zhang, Min Liu, Jingbo Pi, Dawei Guan

**Affiliations:** ^1^Department of Forensic Pathology, School of Forensic Medicine, China Medical University, Shenyang, Liaoning 110001, China; ^2^Institute for Chemical Safety Sciences, The Hamner Institutes for Health Sciences, Research Triangle Park, NC 27709-2137, USA; ^3^Department of Histology and Embryology, School of Basic Medicine, China Medical University, Shenyang, Liaoning 110001, China; ^4^Department of Infectious Diseases, The First Affiliated Hospital, China Medical University, Shenyang, Liaoning 110001, China

## Abstract

Human exposure to inorganic arsenic leads to various dermal disorders, including hyperkeratosis and skin cancer. Curcumin is demonstrated to induce remarkable antioxidant activity in a variety of cells and tissues. The present study aimed at identifying curcumin as a potent activator of nuclear factor erythroid 2-related factor 2 (NRF2) and demonstrating its protective effect against inorganic arsenite- (iAs^3+^-) induced cytotoxicity in human keratinocytes. We found that curcumin led to nuclear accumulation of NRF2 protein and increased the expression of antioxidant response element- (ARE-) regulated genes in HaCaT keratinocytes in concentration- and time-dependent manners. High concentration of curcumin (20 **μ**M) also increased protein expression of long isoforms of NRF1. Treatment with low concentrations of curcumin (2.5 or 5 **μ**M) effectively increased the viability and survival of HaCaT cells against iAs^3+^-induced cytotoxicity as assessed by the MTT assay and flow cytometry and also attenuated iAs^3+^-induced expression of cleaved caspase-3 and cleaved PARP protein. Selective knockdown of *NRF2* or *KEAP1* by lentiviral shRNAs significantly diminished the cytoprotection conferred by curcumin, suggesting that the protection against iAs^3+^-induced cytotoxicity is dependent on the activation of NRF2. Our results provided a proof of the concept of using curcumin to activate the NRF2 pathway to alleviate arsenic-induced dermal damage.

## 1. Introduction

Arsenic is a natural element ubiquitous in the environment. Chronic human exposure to inorganic arsenic (iAs) induces various skin lesions, including Bowen's disease, hyperkeratosis, and skin cancers [1–4]. Our previous studies reveal that oxidative stress occurs in response to inorganic arsenite (iAs^3+^) exposure [5–8], which may partly account for the dermal toxicity of iAs^3+^, including hyperkeratosis and carcinogenesis.

Nuclear factor erythroid 2-related factors (NRFs) are a family of transcription factors that regulate the cellular adaptive response to oxidative stress through the *cis*-regulating antioxidant response element (ARE). Many ARE-dependent genes are important in maintaining the cellular redox homeostasis and limit oxidative damage. Under normal conditions, NRF1 is targeted to the endoplasmic reticulum [9], whereas NRF2, constitutively expressed at a low level, is primarily in the cytoplasm and mainly controlled by the Kelch-like ECH-associated protein 1 (KEAP1) through ubiquitination and proteasomal degradation [10]. Upon oxidative stress, NRF2 and/or NRF1 dimerize with small MAF or other bZIP proteins in the nucleoplasm, and then the heterodimer binds to the AREs in the promoter regions of various detoxifying and antioxidative stress response genes, such as NADPH: quinone oxidoreductase 1 (*NQO1*), glutamate cysteine ligase catalytic (*GCLC*) and regulatory (*GCLM*) subunits, and heme oxygenase-1 (*HMOX-1*). Thus, NRF2 and/or NRF1-mediated adaptive antioxidant response plays important roles against oxidative/electrophilic stress and in chemical detoxification. Our previous studies demonstrated that NRF2, NRF1, and KEAP1 contribute to the coordinated regulation of antioxidant and detoxification enzyme expression and protect cells from arsenic-induced apoptosis and cytotoxicity in human HaCaT cells [6–8, 11]. Therefore, enhancing the NRF2-dependent adaptive response through chemoprevention holds the promise of conferring protection against toxicity and carcinogenicity induced by iAs^3+^.

Curcumin is a polyphenol natural product isolated from the rhizome of *Curcuma longa*. For centuries, curcumin has been used in some medicinal preparations or as a food-coloring agent. Extensive *in vitro* and *in vivo* studies demonstrated that curcumin has a number of biological and pharmacological activities, such as anti-inflammatory, antioxidant, antimutagenic, and anticarcinogenic activities [12–14]. The effects of curcumin have been extensively investigated in liver cells [15], human lymphocytes [16], endothelial cells [17], renal epithelial cells [18], astrocytes [19], and murine splenocytes [20]. The protective effect of curcumin owing to its antioxidant property by inducing NRF2-mediated antioxidant and detoxicating enzymes has been demonstrated [21, 22]. Numerous studies have provided evidence that curcumin protects against iAs^3+^-exerted neurotoxicity, genotoxicity and DNA damage *in vivo* and *in vitro* [20, 23–27]. Thus, with its substantial antioxidant property, circumin can combat the adverse effects of arsenic in a variety of experimental settings and epidemiological surveys.

However, the role of curcumin in regulating NRF2 and its target genes in human keratinocytes and whether curcumin protects against iAs^3+^-induced cytotoxicity in these cells are not clear. In the present study, we confirmed curcumin as a potent NRF2 activator and investigated the NRF2-dependent protective role of curcumin against iAs^3+^-induced cytotoxicity and apoptosis in human HaCaT cells. Our findings have important implications not only for understanding the role of curcumin against iAs^3+^-induced cytotoxicity in human keratinocytes but also for developing preventive and/or corrective strategies against chronic arsenicosis, including arsenic-induced skin disorders.

## 2. Materials and Methods

### 2.1. Cell Culture and Experimental Reagents

HaCaT cells were cultured in Dulbecco's modified Eagle's medium (DMEM) supplemented with 10% fetal bovine serum (FBS), 100 U penicillin/mL, and 100 *μ*g streptomycin/mL as described previously [6]. Cultures were maintained at 37°C in a humidified 5% CO_2_ atmosphere. Culture media, FBS, and supplements were purchased from Invitrogen (Carlsbad, CA, USA). Sodium arsenite and curcumin were obtained from Sigma (St. Louis, MO, USA). 

### 2.2. Lentiviral-Based shRNA Transduction

MISSION shRNA lentiviral particles were obtained from Sigma. Transduction of HaCaT cells with lentiviral-based shRNAs targeting *NRF2* (SHVRS-NM_006164), *KEAP1* (SHVRS-NM_012289), or scrambled nontarget negative control (SHC002V) was performed and confirmed as described previously [7, 8]. Cells were maintained in medium containing 1.0 *μ*g/mL of puromycin.

### 2.3. Antioxidant Response Element (ARE) Reporter Assay

Cignal Lenti ARE reporter transduction of HaCaT cells was performed as described previously [8]. Cells were grown to ~90% confluence and subcultured in medium containing 1.0 *μ*g/mL of puromycin. The luciferase activity was measured by Luciferase Reporter Assay System (E1960, Promega, Madison, WI, USA) according to the manufacturer's protocol. The luciferase activity was normalized to cell viability which was determined using a Non-Radioactive Cell-Proliferation Assay Kit (G5430, Promega).

### 2.4. Acute Cytotoxicity Assay

A minimum of 5 replicates of 10,000 cells per well were plated in 96-well plates and allowed to adhere to the plate for 24 hr, at which time the media were removed and the cells were treated with medium containing curcumin and/or iAs^3+^. Cells were then incubated for indicated time and cell viability was determined using Non-Radioactive Cell-Proliferation Assay Kit as detailed previously [7, 8].

### 2.5. Western Blot Analysis

Protein isolation from whole-cell lysates and determination of protein concentration were conducted with BCA kit according to the manufacturer's protocol (Beyotime, P0010, Shanghai, China). For immunoblot analysis, 50 *μ*g protein was run on an 8% or 12% Tris-Glycine gel and blotted to PVDF membrane. The membrane was blocked in 5% nonfat milk at room temperature (RT) for 2 hr, then it was incubated with primary antibodies (Ab) at 4°C overnight followed by treatment with horseradish peroxidase-conjugated secondary Ab at RT for 2 hr. Protein expression was detected by Chemiluminescence Luminol Reagent (sc-2048, Santa Cruz Biotechnology, Inc., Santa Cruz, CA, USA). Immunoblotting was performed by using Abs against the following antigens: NRF2 (1 : 500, sc-13032, Santa Cruz Biotechnology, Inc.), NRF1 (1 : 500, sc-13031, Santa Cruz Biotechnology, Inc.), KEAP1 (1 : 500, sc-15246, Santa Cruz Biotechnology, Inc.), HMOX-1 (1 : 500, sc-136960, Santa Cruz Biotechnology, Inc.), Cleaved caspase-3 (1 : 1000, #9664, Cell Signaling Technology, MA, USA), PARP (1 : 1000, #9542, Cell Signaling Technology), and *β*-actin (1 : 2000, A1978, Sigma).

### 2.6. Immunostaining

Fluorescence immunostaining was performed as described previously [6]. Briefly, cells were grown on glass cover slips in six-well plates for 48 hr. Then, cells were washed with PBS and fixed for 15 min at RT in 3% (v/v) formaldehyde. After being washed, in PBS, cells were permeabilized in 1% (v/v) Triton X-100 in PBS, washed and incubated with 10% goat serum (ZLI-9021, ZSGB-Bio, Beijing, China) in PBS for 1 hr at RT. Cells were first treated with NRF2 antibody overnight at 4°C and subsequently with goat anti-rabbit IgG-CFL 488 (sc-362262, Santa Cruz Biotechnology, Inc.) for 1 hr at RT. After being washed with PBS, the cover slips were mounted with the Prolong Gold antifade reagent (P36930, Molecular Probes, Inc., Eugene, OR, USA) on microscope slides, and immunostaining was examined by using Leica DM4000 B Fluorescence microscope (Leica Microsystems CMS GmbH, Wetzlar, Germany).

### 2.7. Quantitative Real-Time RT-PCR Analysis

Total RNA was isolated with TRIzol (10296-028, Life Technologies) and then was subjected to cleanup by using RNase-Free DNase Set and RNeasy Mini kit (Qiagen, Valencia, CA, USA). Quantitative real-time RT-PCR was performed as described previously [7, 8]. SYBR Green PCR master mix was purchased from Applied Biosystems (Carlsbad, CA, USA). Primers (sequences are shown in Supplemental Material, Table 1) (Supplementary Material available online at http://dx.doi.org/10.1155/2013/412576) were designed by using Primer Express 4 (Applied Biosystems) and synthesized by MWG-BIOTECH Inc. (High Point, NC, USA). Real-time fluorescence detection was carried out by using an ABI PRISM 7900 HT Fast Real-Time PCR System (Applied Biosystems).

### 2.8. Cell Death Assessment by Flow Cytometry

HaCaT cells were seeded in a six-well plate and grown to approximately 70% confluence. Cells were treated with various concentrations of curcumin for a total of 26 hr. At the end of the 6th hr, iAs^3+^ was added for the remaining 20 hr. Floating and attached cells were harvested for apoptosis analysis. Apoptotic and necrotic cells were analyzed by flow cytometry (Muse Cell Analyser, Merck Millipore, Billerica, MA, USA) by using the Muse Annexin V & Dead Cell Kit (MCH100105, Merck Millipore). For each sample, approximately 10,000 cells were examined each time and the percentage of apoptotic and necrotic cells was calculated from experiments run in triplicate by statistical analysis of the various dot plot using Muse 1.1.2 analysis software (Merck Millipore).

### 2.9. Statistical Analyses

All statistical analyses were performed by using Graphpad Prism 5 (GraphPad Software, San Diego, CA, USA), with *P* < 0.05 taken as significant. Data are expressed as mean ± SD. Statistical analyses to evaluate the time- and concentration-dependent effects of curcumin exposure on gene expression and cell viability were performed by using two-way ANOVA with Bonferroni post hoc testing. Statistical analyses to evaluate the protective effect of curcumin on iAs^3+^-induced cytotoxicity were carried out by using one-way ANOVA with Tukey's multiple comparison test. 

## 3. Results

### 3.1. Cytotoxicity and ARE-Luciferase Activity Induced by Curcumin

**Figure 1:**
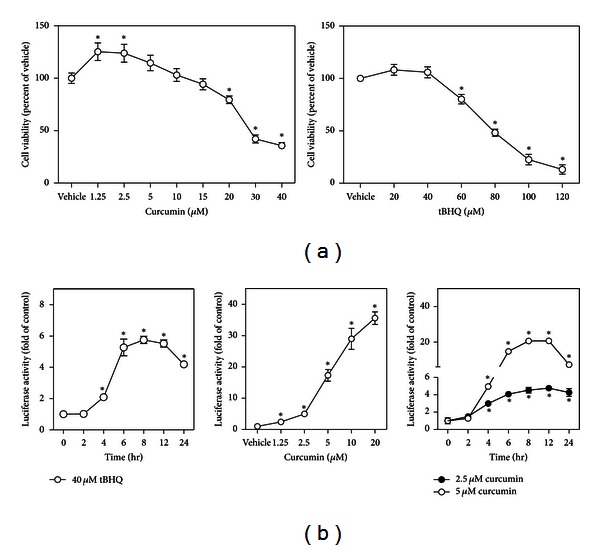
Curcumin activates ARE in HaCaT cells. (a) Cytotoxicity of curcumin and tBHQ. HaCaT cells were exposed to different concentrations of curcumin or tBHQ for 24 hr, and then cell viability was measured by MTT assay. Data are expressed as mean ± SD. *n* = 6. *P < 0.05 versus vehicle (medium). (b) Curcumin activates ARE in a concentration- and time-dependent manner. Cultured HaCaT cells stably transduced with the ARE-luciferase reporter were exposed to 40 *μ*M tBHQ for indicated period of time as a positive control (left panel); then ARE activation was detected in the condition of different concentrations of curcumin for 6 hr (middle panel) or 2.5 and 5 *μ*M of curcumin for indicated period of time (right panel). Values shown are mean ± SD. *n* = 6. *P < 0.05 versus vehicle (medium).

After HaCaT cells were exposed to various concentrations of curcumin for 24 hr, curcumin at 1.25~5 *μ*M significantly increased the viability of HaCaT cells compared to control, while there was a concentration-dependent decrease in cell viability at 10 *μ*M or higher. The LC_50_ of curcumin for 24 hr exposure was 27.33 ± 1.53 *μ*M (Figure 1(a), left panel).

To evaluate the potential activation of the antioxidant response pathway by curcumin in HaCaT cells, cells stably transduced with the ARE-luciferase reporter were used. These cells were responsive to noncytotoxic concentration (40 *μ*M) of NRF2 activator tBHQ-induced ARE activation in a time-dependent fashion (Figure 1(a), right panel, and Figure 1(b), left panel), confirming that the cells are responsive to NRF2 activation. As shown in Figure 1(b) (middle and right panels), curcumin concentration and time dependently increased the activity of ARE-luciferase reporter in HaCaT cells. 

### 3.2. Curcumin Increased NRF2 Protein Expression and Induced the Adaptive Antioxidant Response

**Figure 2:**
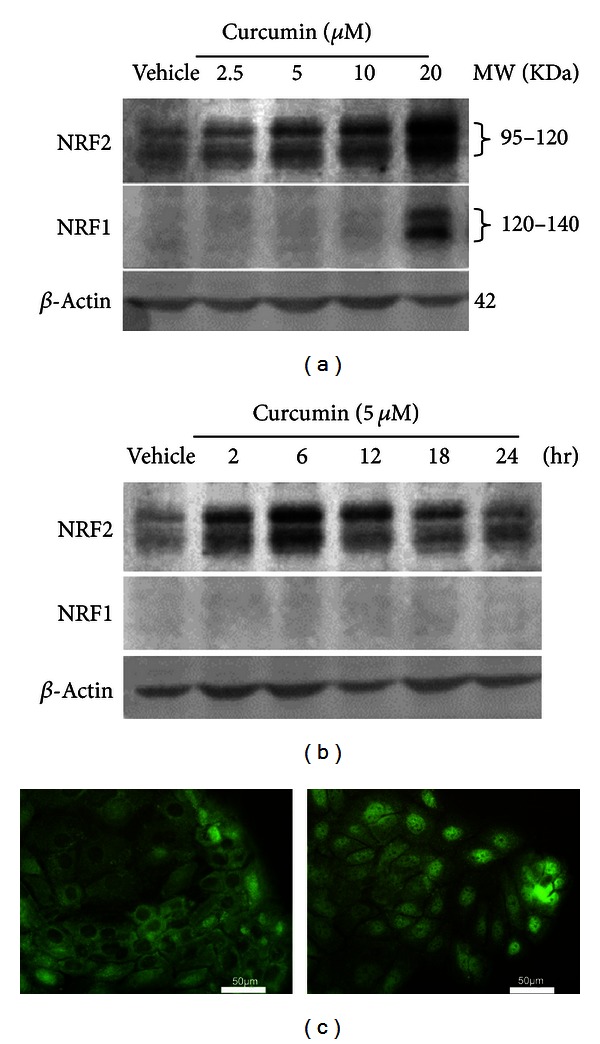
Effects of curcumin on protein expression of NRF2 and NRF1 in HaCaT cells (a) and (b). Representative images of western blot. HaCaT cells were exposed to indicate concentrations of curcumin for 6 hr (a) or 5 *μ*M curcumin for the indicated period of time (b). Whole-cell lysates (50 *μ*g protein) were separated on 8% Tris-Glycine gels and detected using anti-NRF2 or anti-NRF1. *β*-Actin was used as a loading control. Vehicle, medium. (c) Immunofluorescence staining of NRF2. HaCaT cells were treated with vehicle (left) or 5 *μ*M curcumin (right) for 6 hr.

In response to a 6 hr exposure to curcumin, the protein expression of NRF2 was increased in a concentration-dependent manner (Figure 2(a)). In response to 5 *μ*M curcumin treatment, NRF2 protein was elevated quickly and peaked 2~6 hr (Figure 2(b)). These results confirmed that curcumin is a potent NRF2 activator in human HaCaT cells. Interestingly, protein expression of NRF1 was elevated only at a higher concentration of curcumin (20 *μ*M) (Figure 2(a)), suggesting that NRF1 can probably be activated only at more toxic conditions. Cell immunostaining showed that NRF2 was mainly localized in the cytoplasm in untreated cells (Figure 2(c), left panel) but accumulated in the nucleus after exposure to curcumin for 6 hr (Figure 2(c), right panel).

**Figure 3:**
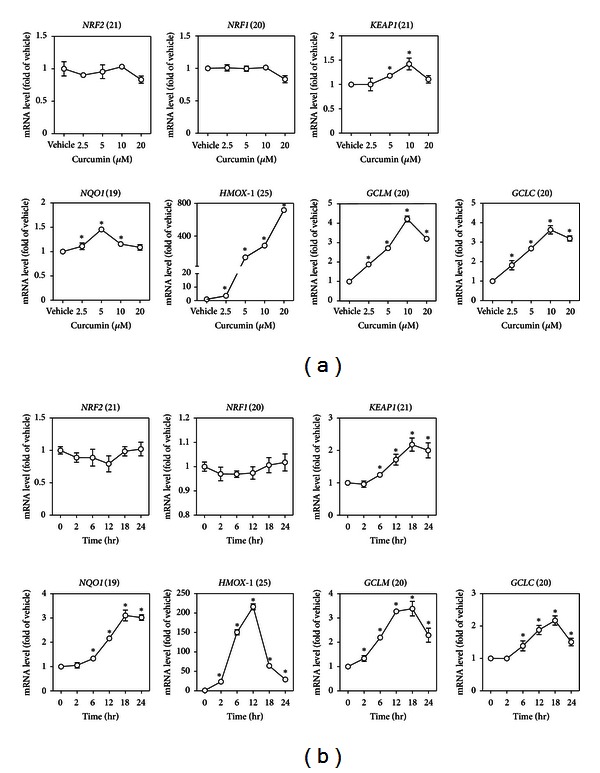
mRNA expression of *NRF2*, *NRF1*, *KEAP1,* and some *ARE*-dependent genes induced by curcumin in HaCaT cells. (a) Concentration response of curcumin-induced gene expression. Cells were exposed to different concentrations of curcumin for 6 hr. (b) Time course of gene expression induced by 5 *μ*M curcumin. The number in parentheses after each gene name is the Ct (cross-threshold) value of that gene in HaCaT cells treated with vehicle (medium). Values are mean ± SD. *n* = 3. *P < 0.05 versus vehicle.

Since curcumin augmented the ARE activity of the luciferase reporter (Figure 1(b)), we next sought to confirm the result with endogenous* ARE*-dependent genes. As expected, mRNAs of *NQO1*, *HMOX1*, *GCLC,* and *GCLM* were induced significantly by curcumin in a concentration- and time-dependant manner (Figures 3(a) and 3(b), bottom panels). The mRNA expression of *NRF2* and *NRF1* decreased slightly at a high concentration of curcumin and did not change significantly over time (Figures 3(a) and 3(b), upper panels), suggesting that* NRF2* and *NRF1* were primarily posttranscriptionally regulated. Interestingly, the mRNA expression of *KEAP1 *was also induced by curcumin, suggesting a potential feedback from NRF2 to KEAP1. Our results demonstrate that curcumin is able to induce the NRF2 pathway and its target genes.

### 3.3. Curcumin Protected against iAs^3+^-Induced Cytotoxicity

**Figure 4:**
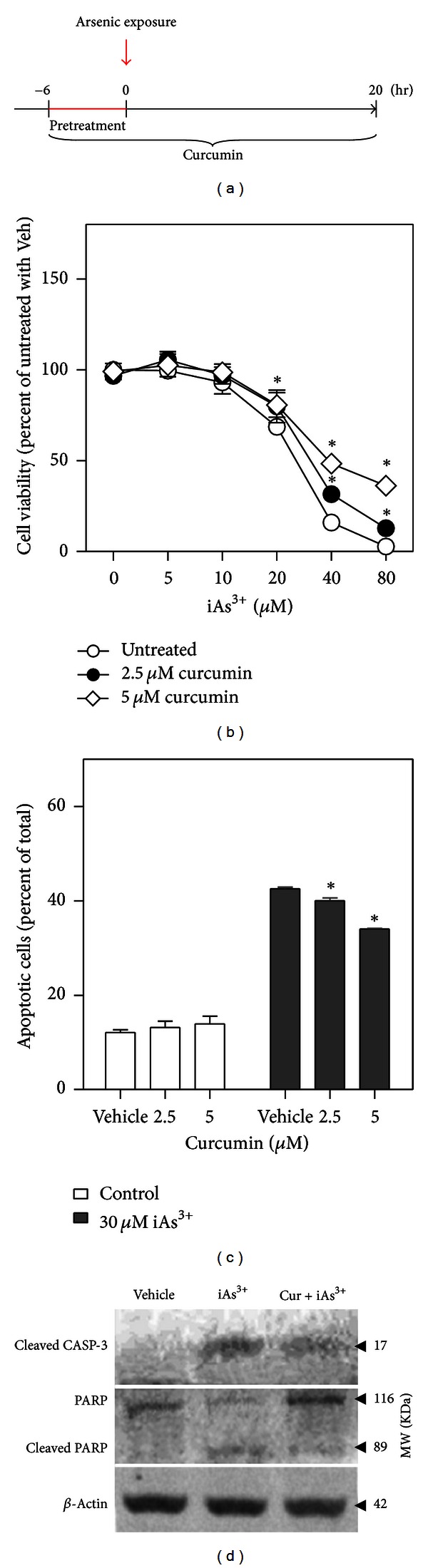
Curcumin treatment protects HaCaT cells against iAs^3+^-induced cytotoxicity. (a) Protocols for curcumin treatment and arsenic exposure in HaCaT cells. Confluence cells were either treated or not treated with curcumin for a total of 26 hr. After the first 6 hr (pretreatment), arsenic was added for the remaining 20 hr. (b) Effect of curcumin treatment on iAs^3+^-induced cytotoxicity. Cell viability was measured by MTT assay. Values are mean ± SD. *n* = 6. *P < 0.05 versus untreated with the same iAs^3+^ exposure. Veh, vehicle. (c) Effect of curcumin treatment on iAs^3+^-induced apoptosis. Apoptotic cells were determined by flow cytometry. Annexin V-positive cells were quantified as apoptotic cells. *n* = 3. *P < 0.05 versus iAs^3+^ exposure without curcumin treatment. (d) Immunoblotting of cleaved caspase-3 (CASP-3), PARP, and cleaved PARP. Vehicle, medium; iAs^3+^, cells exposed to 30 *μ*M of iAs^3+^ for 20 hr; Cur + iAs^3+^, cells treated with 5 *μ*M curcumin for 26 hr and exposed to 30 *μ*M of iAs^3+^ for 20 hr. Whole cell lysates were used for analysis and *β*-ACTIN was used as loading control.

To determine the protective effect of curcumin on iAs^3+^-induced cytotoxicity, noncytotoxic concentrations of curcumin (2.5 and 5 *μ*M, Figure 1(a)) were used. As shown in Figure 4, HaCaT cells were pretreated with 2.5 *μ*M or 5 *μ*M curcumin for 6 hr. Subsequently, the cells were exposed to 30 *μ*M of iAs^3+^ for 20 hr in the continued presence of curcumin (Figure 4(a)), after which cell viability and apoptosis were measured. Compared with untreated cells, treatment with curcumin caused a significant increase in cell viability in response to iAs^3+^ (Figure 4(b)). In addition, flow cytometry measurement with Annexin V-FITC and PI double staining showed that exposing cells to 30 *μ*M iAs^3+^ for 20 hr dramatically increased the percentage of apoptotic cells, whereas 2.5 *μ*M or 5 *μ*M curcumin reduced apoptotic cell death (Figure 4(c)). Apoptosis was not affected by treatment with 2.5 *μ*M or 5 *μ*M curcumin alone. To further confirm the protective effect of curcumin against iAs^3+^-induced cytotoxicity, we examined the levels of cleaved caspase-3 and PARP protein. Immunoblotting showed that iAs^3+^ at 30 *μ*M increased the levels of cleaved caspase-3 and cleaved PARP, while treatment with curcumin (5 *μ*M) attenuated the increase (Figure 4(d)). Together, these results demonstrated that curcumin at low noncytotoxic concentrations is able to protect cells from iAs^3+^-induced cellular toxicity.

### 3.4. The Protective Effect of Curcumin on iAs^3+^-Induced Cytotoxicity and Apoptosis Is Dependent on NRF2 Activation

**Figure 5:**
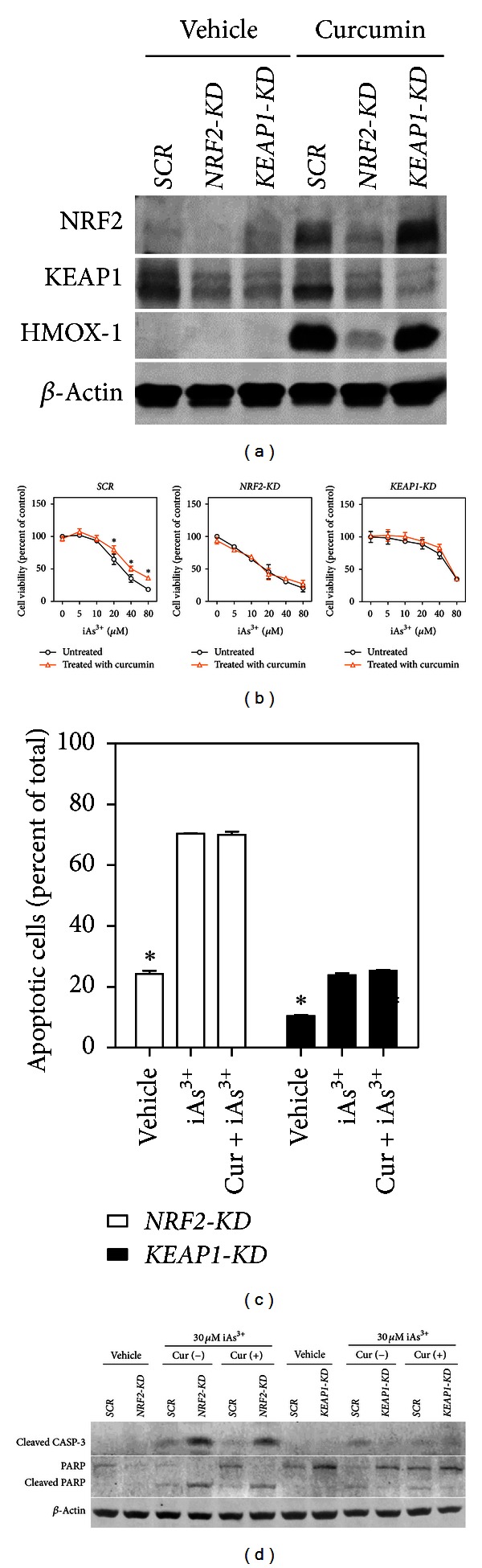
The protective effect of curcumin on iAs^3+^-induced cytotoxicity and apoptosis is dependent on NRF2 activation in HaCaT cells. Protocols for curcumin treatment and arsenic exposure are the same as Figure 4(a). (a) The protein level of NRF2, KEAP1, and HMOX-1 under basal and curcumin-treated condition in scramble, *NRF2-KD,* and *KEAP1-KD* cells. Cells were treated with vehicle or 20 *μ*M curcumin for 6 hr. Whole-cell lysates were separated on 4–12% Tris-Glycine gels. Vehicle, medium. (b) The effect of curcumin treatment on iAs^3+^-induced cytotoxicity in Scramble, *NRF2-*KD and *KEAP1*-KD cells. Cell viability was measured by MTT assay. Values are mean ± SD. *n* = 6. *P < 0.05 versus curcumin-untreated with the same iAs^3+^ exposure. (c) Effect of curcumin treatment on iAs^3+^-induced apoptosis in *NRF2*-KD and *KEAP1*-KD cells. Apoptotic cells were determined by flow cytometry. Annexin V-positive cells were quantified as apoptotic cells. *n* = 3. (d) Immunoblotting of cleaved caspase-3, PARP, and cleaved PARP. Vehicle, medium; iAs^3+^, cells exposed to 30 *μ*M of iAs^3+^ for 20 hr; Cur + iAs^3+^, cells treated with 5 *μ*M curcumin for 26 hr and exposed to iAs^3+^ for 20 hr. Whole-cell lysates were used for analysis and *β*-actin was used as a loading control.

We proposed that the protection against the cytotoxicity of iAs^3+^ by curcumin in human HaCaT cells is owing to the activation of NRF2. To study the mechanism, the effects of curcumin on iAs^3+^-induced cytotoxicity in HaCaT cells with stable knockdown (KD) of *NRF2* or *KEAP1* were examined. The silencing efficiency of the constructs was confirmed by immunoblot under basal and curcumin-treated condition (Figure 5(a)), at the same time, the protein level of HMOX-1, an NRF2-specific downstream gene, was also detected (Figure 5(a)). As expected, the protection by curcumin against iAs^3+^ was obvious in scramble (SCR) cells (Figure 5(b), left panel). In *NRF2*-KD cells, treatment with 5 *μ*M curcumin offered no protection against iAs^3+^ as compared with cells exposed to iAs^3+^ alone (Figure 5(b), midpanel). Interestingly, curcumin did not offer further protection against iAs^3+^ in *KEAP1*-KD cells either (Figure 5(b), right panel). This is likely because in these cells NRF2 was already fully activated due to the lacking of *KEAP1*, and thus maximal protection against iAs^3+^ was already in place, as indicated by the dramatic right-ward shift of the response curve in untreated cells. Therefore, treatment with circumin did not provide additionally activated NRF2 and hence no further protection. The fraction of apoptotic cells, as revealed by flow cytometry (Figure 5(c)) and cleaved caspase-3/cleaved PARP expression (Figure 5(d)), showed no difference between curcumin-treated and untreated cells with either *NRF2*-KD or *KEAP1*-KD. Our data demonstrate that the cytoprotection provided by curcumin requires activation of the NRF2 pathway.

## 4. Discussion

NRF2 plays a pivotal role in directly regulating many antioxidant and detoxification enzyme genes via AREs in gene promoters. The important role of NRF2 in chemoprevention and cellular defense has been clearly demonstrated in *NRF2*-null mice which are susceptible to both oxidative and carcinogenic insults [28–31]. Accumulating evidence from both animal models and human epidemiological studies has shown that many naturally occurring phytochemicals, including sulforaphane, epigallocatechin-3-gallate (EGCG), curcumin, and oridonin, possess chemopreventive potential by inducing NRF2-mediated antioxidant/detoxification enzymes [32–34].

Curcumin has been used for chemoprevention and treatment of various skin lesions, such as scleroderma, psoriasis, skin cancer, and wound healing [35–37]. It has been shown that curcumin activated NRF2 in several cell types [21, 32, 38] and exerted a cytoprotective effect through transcriptional induction of phase II enzymes, such as glutathione transferase, NQO1, HMOX-1, GCLC, and GCLM in certain human cancers, skin lesions, and neurodegenerative diseases [39, 40]. However, whether curcumin is a potent NRF2/1 activator in human HaCaT cells has not been demonstrated previously. Our present study indicates that curcumin induced NRF2 protein nuclear accumulation in a time- and concentration-dependent manner, which is unlikely to be attributed to an increase in mRNA expression, confirming a previous report [41]. Though curcumin disrupted the NRF2-KEAP1 complex, leading to increased NRF2 occupancy of AREs [42], or indirectly stimulated the phosphorylation of NRF2 at serine and/or threonine residues which may facilitate its nuclear accumulation [21], the exact mechanism by which curcumin activates NRF2 needs further investigation. Furthermore, research using relative high concentrations of curcumin (10~25 *μ*M) on cultured cells showed that curcumin upregulated phase II enzymes especially HMOX-1 [41, 43], while, in our experiment, curcumin was found to readily induce *NQO1*, *HMOX-1*, *GCLC,* and *GCLM* genes at even lower concentrations.

Curcumin has been found to affect the structure and function of cellular membrane, mimic typical events occurring during apoptosis [44], and induce apoptosis of epidermal cells at the concentration of 12.5 or 25 *μ*M [41]. When HaCaT cells were treated in combination with UV or radiation, they showed increased apoptosis [45, 46]. In our studies, acute exposure to curcumin at low concentrations has no effect on the apoptotic rate, while it increased viability against iAs^3+^-induced cytotoxicity. 

Arsenic, a ubiquitous environmental element, causes dermal toxicity [4, 47]. Our previous studies [6–8] demonstrated that the NRF2 and NRF1 signaling pathways can be activated by iAs^3+^ in human HaCaT cells, suggesting that NRF2 and NRF1 may be involved in the pathogenesis of arsenic-induced skin cancer and hyperkeratosis. Furthermore, numerous studies show that the NRF2-mediated stress response program is activated in early tumor development, and oncogene activities are coupled with NRF2 activation, thereby providing malignant cells a survival and growth advantage [48–50]. However, activating the NRF2-dependent protective pathway has also proved to be beneficial in reducing arsenic-induced toxicity in human bladder urothelial cells [51]. Our previous study showed that stable knockdown of NRF2 using shRNA rendered human HaCaT cells more sensitive to iAs^3+^-induced cell death [8], suggesting a potential usage of NRF2 activators for therapeutic and dietary interventions against adverse effects of arsenic. The paradoxical health effects of NRF2 activated by a specific chemical agent were mainly determined by the balance between the induction of the NRF2 defense response and the otherwise adverse outcomes elicited by the agent. Therefore, the agent used for cytoprotection should be preferred at low concentrations without eliciting tangible cytotoxicity. In the present study, treatment with curcumin counteracted iAs^3+^-induced cell damage through activating NRF2, as demonstrated by MTT, apoptosis, and apoptotic-executive protein expression assays. Our cell-based study supports the notion that curcumin can be used as a chemopreventive agent. Low-concentration curcumin specifically targets NRF2-induced cellular antioxidant defense and has an important role in maintaining homeostasis in epidermis [52]. Although the finding that treatment with curcumin led to the activation of NRF2 and protected HaCaT cells against the acute cytotoxicity of iAs^3+^ is consistent with the result in hepatocytes with sulforaphane [53], the suppression of oxidative stress and/or reduction of cellular accumulation of arsenic in HaCaT cells needs further investigation to illustrate the underlying mechanisms. At noncytotoxic concentrations, curcumin had no effect on NRF1 protein expression; thus, the protective effect of curcumin in these concentrations is mainly NRF2 dependent. NRF1 is an essential gene during development [54], and the 120-kD isoform of NRF1 is glycosylated and located in the ER [55], which mediates antioxidant defense response against arsenic-induced cytotoxicity in human keratinocytes [7]. Our present results reveal that the protein expression of long isoforms of NRF1 is accumulated by high concentrations of curcumin, which is consistent with the distinctive role of NRF1; that is, NRF1 may offer protection in more severe stress conditions or provide additional protection when the antioxidant capacity provided by NRF2 is exhausted. This work provides a proof of concept of using curcumin to activate the NRF2 pathway to alleviate arsenic-induced damage and suggests that its chemopreventive potential requires optimization of dose.

## 5. Conclusion

Curcumin functions as a chemopreventive compound at low concentrations against arsenic-induced damage, which is mediated through activating the NRF2 cytoprotective pathway.

## Supplementary Material

Primer sequences for Real-time RT-PCR used in this study. The candidated genes name, genebank accession, position and sequences are given in Supplemental Table 1.

## Abbreviation

Ab: Antibody
AREs: Antioxidant response elements
CASP-3: Caspase-3
Cur: Curcumin
DMEM: Dulbecco's modified Eagle's medium
ECL: Enhanced chemiluminescence
EGCG: Epigallocatechin-3-gallate
FBS: Fetal bovine serum
FITC: Fluorescein-4-isothiocyanate
GCLC: Glutamate cysteine ligase catalytic subunits
GCLM: Glutamate cysteine ligase regulatory subunits
HMOX-1: Heme oxygenase-1
iAs^3+^: Arsenite
KD: Knockdown
KEAP1: Kelch-like ECH-associated protein 1
MTT: 3-(4,5)-Dimethylthiahiazo(-z-y1)-3,5-di-phenytetrazoliumromide
NQO1: NADPHquinone oxidoreductase 1
NRF1: Nuclear factor erythroid 2-related factor 1
NRF2: Nuclear factor erythroid 2-related factor 2
RT: Room temperature
RT-PCR: Reverse transcription-polymerase chain reaction
SCR: Scramble
tBHQ: *tert*-butylhydroquinone
Veh: Vehicle.

## References

[1] Pi J, Kumagai Y, Sun G (2000). Decreased serum concentrations of nitric oxide metabolites among Chinese in an endemic area of chronic arsenic poisoning in inner Mongolia. *Free Radical Biology and Medicine*.

[2] Yoshida T, Yamauchi H, Fan Sun G (2004). Chronic health effects in people exposed to arsenic via the drinking water: dose-response relationships in review. *Toxicology and Applied Pharmacology*.

[3] Wong SS, Tan KC, Goh CL (1998). Cutaneous manifestations of chronic arsenicism: review of seventeen cases. *Journal of the American Academy of Dermatology*.

[4] IARC Working Group on the Evaluation of Carcinogenic Risks to Humans (2004). Some drinking-water disinfectants and contaminants, including arsenic. *IARC Monographs on the Evaluation of Carcinogenic Risks to Humans*.

[5] Pi J, Yamauchi H, Kumagai Y (2002). Evidence for induction of oxidative stress caused by chronic exposure of Chinese residents to arsenic contained in drinking water. *Environmental Health Perspectives*.

[6] Pi J, Qu W, Reece JM, Kumagai Y, Waalkes MP (2003). Transcription factor Nrf2 activation by inorganic arsenic in cultured keratinocytes: involvement of hydrogen peroxide. *Experimental Cell Research*.

[7] Zhao R, Hou Y, Xue P (2011). Long isoforms of NRF1 contribute to arsenic-induced antioxidant response in human keratinocytes. *Environmental Health Perspectives*.

[8] Zhao R, Hou Y, Zhang Q (2012). Cross-regulations among NRFs and KEAP1 and effects of their silencing on arsenic-induced antioxidant response and cytotoxicity in human keratinocytes. *Environmental Health Perspectives*.

[9] Biswas M, Chan JY (2010). Role of Nrf1 in antioxidant response element-mediated gene expression and beyond. *Toxicology and Applied Pharmacology*.

[10] Hayes JD, McMahon M (2009). NRF2 and KEAP1 mutations: permanent activation of an adaptive response in cancer. *Trends in Biochemical Sciences*.

[11] Aono J, Yanagawa T, Itoh K (2003). Activation of Nrf2 and accumulation of ubiquitinated A170 by arsenic in osteoblasts. *Biochemical and Biophysical Research Communications*.

[12] Zhou H, Beevers CS, Huang S (2011). The targets of curcumin. *Current Drug Targets*.

[13] Mano Y, Usui T, Kamimura H (2006). In vitro inhibitory effects of non-steroidal anti-inflammatory drugs on 4-methylumbelliferone glucuronidation in recombinant human UDP-glucuronosyltransferase 1A9—potent inhibition by niflumic acid. *Biopharmaceutics and Drug Disposition*.

[14] Divya CS, Pillai MR (2006). Antitumor action of curcumin in human papillomavirus associated cells involves downregulation of viral oncogenes, prevention of NFkB and AP-1 translocation, and modulation of apoptosis. *Molecular Carcinogenesis*.

[15] Rukkumani R, Aruna K, Varma PS, Menon VP (2004). Curcumin influences hepatic expression patterns of matrix metalloproteinases in liver toxicity. *The Italian Journal of Biochemistry*.

[16] Wang H, Geng QR, Wang L, Lu Y Curcumin potentiates antitumor activity of L-asparaginase via inhibition of the AKT signaling pathway in acute lymphoblastic leukemia. *Leukemia & Lymphoma*.

[17] Jeong GS, Oh GS, Pae HO (2006). Comparative effects of curcuminoids on endothelial heme oxygenase-1 expression: ortho-methoxy groups are essential to enhance heme oxygenase activity and protection. *Experimental and Molecular Medicine*.

[18] Balogun E, Foresti R, Green CJ, Motterlini R (2003). Changes in temperature modulate heme oxygenase-1 induction by curcumin in renal epithelial cells. *Biochemical and Biophysical Research Communications*.

[19] Scapagnini G, Foresti R, Calabrese V, Giuffrida Stella AM, Green CJ, Motterlini R (2002). Caffeic acid phenethyl ester and curcumin: a novel class of heme oxygenase-1 inducers. *Molecular Pharmacology*.

[20] Khan S, Vala JA, Nabi SU Protective effect of curcumin against arsenic-induced apoptosis in murine splenocytes in vitro. *Journal of Immunotoxicology*.

[21] Farombi EO, Shrotriya S, Na HK, Kim SH, Surh YJ (2008). Curcumin attenuates dimethylnitrosamine-induced liver injury in rats through Nrf2-mediated induction of heme oxygenase-1. *Food and Chemical Toxicology*.

[22] Lele RD (2010). Beyond reverse pharmacology: mechanism-based screening of Ayurvedic drugs. *Journal of Ayurveda and Integrative Medicine*.

[23] Yadav A, Lomash V, Samim M, Flora SJ (2012). Curcumin encapsulated in chitosan nanoparticles: a novel strategy for the treatment of arsenic toxicity. *Chemico-Biological Interactions*.

[24] Biswas J, Roy S, Mukherjee S, Sinha D, Roy M (2010). Indian spice curcumin may be an effective strategy to combat the genotoxicity of arsenic in Swiss albino mice. *Asian Pacific Journal of Cancer Prevention*.

[25] Yadav RS, Shukla RK, Sankhwar ML (2010). Neuroprotective effect of curcumin in arsenic-induced neurotoxicity in rats. *NeuroToxicology*.

[26] Suwannateep N, Wanichwecharungruang S, Haag SF (2012). Encapsulated curcumin results in prolonged curcumin activity in vitro and radical scavenging activity ex vivo on skin after UVB-irradiation. *European Journal of Pharmaceutics and Biopharmaceutics*.

[27] Yadav RS, Sankhwar ML, Shukla RK (2009). Attenuation of arsenic neurotoxicity by curcumin in rats. *Toxicology and Applied Pharmacology*.

[28] McMahon M, Itoh K, Yamamoto M (2001). The cap “n” collar basic leucine zipper transcription factor Nrf2 (NF-E2 p45-related factor 2) controls both constitutive and inducible expression of intestinal detoxification and glutathione biosynthetic enzymes. *Cancer Research*.

[29] Reisman SA, Csanaky II, Aleksunes LM, Klaassen CD (2009). Altered disposition of acetaminophen in Nrf2-null and keap1-knockdown mice. *Toxicological Sciences*.

[30] Brzóska K, Stepkowski TM, Kruszewski M (2011). Putative proto-oncogene pir expression is significantly up-regulated in the spleen and kidney of cytosolic superoxide dismutase-deficient mice. *Redox Report*.

[31] Guo XF, Zhu XF, Zhong GS, Deng BG (2012). Retracted: lapatinib, a dual inhibitor of epidermal growth factor receptor and human epidermal growth factor receptor 2, potentiates the antitumor effects of cisplatin on esophageal carcinoma. *Diseases of the Esophagus*.

[32] Jeong SO, Oh GS, Ha HY (2009). Dimethoxycurcumin, a synthetic curcumin analogue, induces heme oxygenase-1 expression through Nrf2 activation in RAW264.7 macrophages. *Journal of Clinical Biochemistry and Nutrition*.

[33] Nishinaka T, Ichijo Y, Ito M (2007). Curcumin activates human glutathione S-transferase P1 expression through antioxidant response element. *Toxicology Letters*.

[34] Du Y, Villeneuve NF, Wang XJ (2008). Oridonin confers protection against arsenic-induced toxicity through activation of the Nrf2-mediated defensive response. *Environmental Health Perspectives*.

[35] Thangapazham RL, Sharma A, Maheshwari RK (2007). Beneficial role of curcumin in skin diseases. *Advances in Experimental Medicine and Biology*.

[36] Kulac M, Aktas C, Tulubas F (2013). The effects of topical treatment with curcumin on burn wound healing in rats. *Journal of Molecular Histology*.

[37] Heng MCY (2010). Curcumin targeted signaling pathways: basis for anti-photoaging and anti-carcinogenic therapy. *International Journal of Dermatology*.

[38] Yang C, Zhang X, Fan H, Liu Y (2009). Curcumin upregulates transcription factor Nrf2, HO-1 expression and protects rat brains against focal ischemia. *Brain Research*.

[39] Hatcher H, Planalp R, Cho J, Torti FM, Torti SV (2008). Curcumin: from ancient medicine to current clinical trials. *Cellular and Molecular Life Sciences*.

[40] Rushworth SA, Ogborne RM, Charalambos CA, O’Connell MA (2006). Role of protein kinase C *δ* in curcumin-induced antioxidant response element-mediated gene expression in human monocytes. *Biochemical and Biophysical Research Communications*.

[41] Natarajan VT, Singh A, Kumar AA (2010). Transcriptional upregulation of Nrf2-dependent phase II detoxification genes in the involved epidermis of vitiligo vulgaris. *The Journal of Investigative Dermatology*.

[42] Banning A, Deubel S, Kluth D, Zhou Z, Brigelius-Flohé R (2005). The GI-GPx gene is a target for Nrf2. *Molecular and Cellular Biology*.

[43] McNally SJ, Harrison EM, Ross JA, Garden OJ, Wigmore SJ (2007). Curcumin induces heme oxygenase 1 through generation of reactive oxygen species, p38 activation and phosphatase inhibition. *International Journal of Molecular Medicine*.

[44] Kossler S, Nofziger C, Jakab M, Dossena S, Paulmichl M (2012). Curcumin affects cell survival and cell volume regulation in human renal and intestinal cells. *Toxicology*.

[45] Dujic J, Kippenberger S, Hoffmann S (2007). Low concentrations of curcumin induce growth arrest and apoptosis in skin keratinocytes only in combination with UVA or visible light. *The Journal of Investigative Dermatology*.

[46] Chendil D, Ranga RS, Meigooni D, Sathishkumar S, Ahmed MM (2004). Curcumin confers radiosensitizing effect in prostate cancer cell line PC-3. *Oncogene*.

[47] Pi J, He Y, Bortner C (2005). Low level, long-term inorganic arsenite exposure causes generalized resistance to apoptosis in cultured human keratinocytes: potential role in skin co-carcinogenesis. *International Journal of Cancer*.

[48] Perera RM, Bardeesy N (2011). Cancer: When antioxidants are bad. *Nature*.

[49] Costa R, Abdulhaq H, Haq B (2011). Activity of azacitidine in chronic myelomonocytic leukemia. *Cancer*.

[50] Hayes JD, McMahon M (2006). The double-edged sword of Nrf2: subversion of redox homeostasis during the evolution of cancer. *Molecular Cell*.

[51] Wang XJ, Sun Z, Chen W, Eblin KE, Gandolfi JA, Zhang DD (2007). Nrf2 protects human bladder urothelial cells from arsenite and monomethylarsonous acid toxicity. *Toxicology and Applied Pharmacology*.

[52] Marrot L, Jones C, Perez P, Meunier JR (2008). The significance of Nrf2 pathway in (photo)-oxidative stress response in melanocytes and keratinocytes of the human epidermis. *Pigment Cell and Melanoma Research*.

[53] Shinkai Y, Sumi D, Fukami I, Ishii T, Kumagai Y (2006). Sulforaphane, an activator of Nrf2, suppresses cellular accumulation of arsenic and its cytotoxicity in primary mouse hepatocytes. *FEBS Letters*.

[54] Chan JY, Kwong M, Lu R (1998). Targeted disruption of the ubiquitous CNC-bZIP transcription factor, Nrf-1, results in anemia and embryonic lethality in mice. *The EMBO Journal*.

[55] Zhang Y, Hayes JD (2010). Identification of topological determinants in the N-terminal domain of transcription factor Nrf1 that control its orientation in the endoplasmic reticulum membrane. *Biochemical Journal*.

