# Supplementary material for: Curcumin Protects Human Keratinocytes against Inorganic Arsenite-Induced Acute Cytotoxicity through an NRF2-Dependent Mechanism
Source: Oxid Med Cell Longev. 2013 Apr 21;2013:412576. doi: 10.1155/2013/412576 (PMC3654359; doi:10.1155/2013/412576)
Supplement: Supplementary file 1 — Primer sequences for Real-time RT-PCR used in this study. The candidated genes name, genebank accession, position and sequences are given in Supplemental Table 1. [file 412576.f1.pdf]

Supplemental Material,

Table 1:

Primer sequences for Real-time RT-PCR

**Gene name**	**GeneBank Accession**	**Position**	**Forward (5' - 3')**	**Reverse (5' - 3')**
NRF1	NM_003204	4172-4259	GCCCTGTTTCACTTATAGGGTCTAGA	GGCAAAGAGAACATTTAGCAGCTT
NRF2	NM-006164	398-488	AACCAGTGGATCTGCCAACTACTC	CTGCGCCAAAAGCTGCAT
KEAP-1	NM_012289	2257-2342	CCTCTGGCCGGGTAATAGG	CCCCTCCCAGGTATCCAAGA
NQO1	NM_000903	2154-2264	ACTGCCCTCTTGTGGTGCAT	GCTCGGTCCAATCCCTTCAT
HMOX-1	NM_002133	1216-1294	GCCTGGAAGACACCCTAATGTG	GGCCGTGTCAACAAGGATACTT
GCLC	NM_001498	1521-1625	GATGCTGTCTTGCAGGGAATG	AGCGAGCTCCGTGCTGTT
GCLM	NM_002061	830-910	ACAGGTAAAACCAAATAGTAACAAAGTTAA	TGTTTAGCAAATGCAGTCAAATCTG
β-ACTIN	X00351	1088-1163	GTCCACCTTCCAGCAGATGTG	GCATTTGCGGTGGACGAT
